# Electrochemical Paper-Based Analytical Device (e-PAD) Using Immobilized Prussian Blue and Antibodies for the Diagnosis of *Leishmania*

**DOI:** 10.1021/acsomega.4c07455

**Published:** 2025-02-12

**Authors:** Maurício Alberto Poletti Papi, Cristiane Kalinke, Carlos R. Soccol, Vanete Thomaz Soccol, Breno C. B. Beirão, Márcio F. Bergamini, Luiz H. Marcolino-Júnior

**Affiliations:** † Laboratory of Electrochemical Sensors (LabSensE), Department of Chemistry, Federal University of Paraná, Curitiba, Paraná 81531-980, Brazil; ‡ Institute of Chemistry, University of Campinas, Campinas, São Paulo 13083-970, Brazil; § Department of Bioprocess Engineering and Biotechnology, Federal University of Paraná, Curitiba, Paraná 81531-980, Brazil; ∥ Graduate Program in Microbiology, Parasitology, and Pathology, Federal University of Paraná (UFPR), CEP, Curitiba, Paraná 81531-980, Brazil

## Abstract

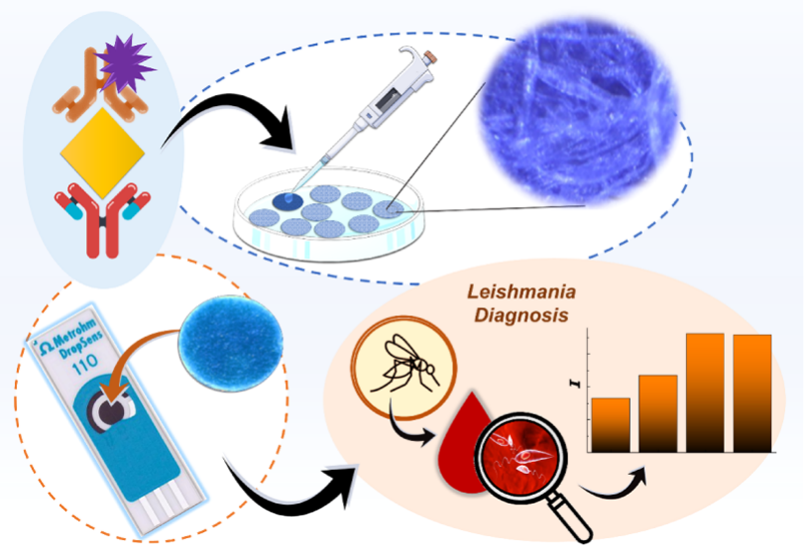

Leishmaniasis is a neglected disease prevalent in remote and economically disadvantaged regions. Its diagnosis can be achieved through various methods, with electroanalysis emerging as an excellent alternative for antigen detection due to its simplicity, sensitivity, and cost-effectiveness. Herein, a qualitative electrochemical paper-based analytical device (e-PAD) using unmodified screen-printed electrodes for the immunoassay of *Leishmania amazonensis* antigens has been developed. The detection is based on a sandwich-type assembly, utilizing two biological elements, one for capture and one for detection, with the target antigen sandwiched between them. Antibodies against *Leishmania amazonensis* and Prussian blue, serving as a redox mediator, were immobilized on the paper substrate. A synthetic peptide was employed as the target to demonstrate the proof-of-concept performance of the device. The formation of the immunocomplex was confirmed using horseradish peroxidase (HRP)-labeled antibodies, enabling the detection of antigen/antibody complexes in the presence of hydrogen peroxide via multiple pulse amperometry (MPA). The immunoassay exhibited good reproducibility (RSD = 5.12%) and selectivity when tested with positive and negative samples. Additionally, the ease of use and low cost of e-PAD enhance its accessibility, making it a valuable tool for the rapid and reliable diagnosis of neglected diseases. This reinforces its relevance as a practical solution in public health, particularly in underserved regions.

## 1 Introduction

Leishmaniasis is a zoonotic, infectious disease caused by the *Leishmania* genus protozoa parasite and transmitted by the bite of *Phlebotomus* female sandflies.^1^ Several species can be reservoirs of the parasite, more specifically, wild animals. However, domestic animals (i.e., dogs) and humans can also be infected.^2^*Leishmania* epidemiology is affected by environmental, migratory, and climatic factors. It is considered a tropical and neglected disease mainly affecting populations in developing or underdeveloped countries, causing up to one million new cases each year.^3^

Different forms of the disease can be found, including cutaneous, mucocutaneous, and visceral leishmaniasis. According to the World Health Organization (WHO), the identification of parasites can be performed by biochemical characteristics (isoenzymes), and the diagnosis can be done by parasitological or serological tests such as enzyme-linked immunosorbent assay (ELISA) or Western blot. However, the methodologies described for the diagnosis of *Leishmania* have several disadvantages linked to testing time, centralization in specialized laboratories, and cost.^4^

The development of new tools with good performance, speed, and feasibility is essential in combating neglected diseases, making it possible to guarantee healthcare accessibility to remote and less well-off regions.^5^ In this way, biosensors, especially electrochemical immunosensors, have been applied as excellent tools for analytical detection in different applications such as biomedical, environmental, food, and diagnosis of infectious diseases, among others.^6,7^ Electrochemical immunosensors are devices that transduce the interaction between antigens and antibodies into measurable electrical signals. They have been widely used in the diagnosis of various diseases, including hepatitis B and C,^8,9^ influenza,^10^ dengue,^11,12^ Zika,^13^ HIV,^14,15^ SARS-CoV-2^16,17^ leishmaniasis,^5,18^ tuberculosis,^19^ among others. These devices offer several advantages over traditional analytical methods, including low cost, portability, miniaturization, ease of use, on-site monitoring, good selectivity, and sensitivity. Despite these advantages, conventional electrochemical systems still face challenges related to nonspecific adsorption of molecules, interference from the sample matrix, and issues with stability and reproducibility in the immobilization of recognition elements, which can significantly affect assay reliability. In addition to these problems, there is difficulty in scaling and regenerating the sensor for repeated use, especially if the immobilized recognition elements are sensitive to the regeneration conditions. Therefore, there is the alternative of not modifying the electrode surface but rather carrying out a bioassay by immobilizing the species (recognition sites and mediators) on more accessible and cheaper platforms, such as paper.

Paper-based devices offer notable advantages, such as disposability and low production costs, making them stand out in various applications. They are compatible with a wide range of biomolecules and offer benefits such as long shelf life, versatility, and the ability to operate with minimal sample quantities. Moreover, paper can be effectively utilized for the immobilization of biomolecules, either within the cellulose matrix or through functionalization of the paper itself, enhancing immobilization efficiency.^20,21^ An interesting approach is achieved by combining the use of electrochemical methods, which offer several advantages to paper-based devices. Paper-based analytical devices integrated with electrochemical detection (e-PADs) have gained popularity due to their high sensitivity, ease of application, and affordability.^22,23^

This work describes the evaluation of an electrochemical immunoassay based on e-PADs, focusing on content recognition sites and immobilized redox mediators. The paper was assembled over an unmodified screen-printed electrode, providing an easy-to-use alternative for the rapid qualitative diagnosis of leishmaniasis in infected patients.

Many immunosensors described in the literature have been designed to provide a quantitative response, which is often highly desirable. However, qualitative sensors offer a simpler binary or categorical output, indicating the presence or absence of a specific target analyte rather than its precise concentration.^6,7^ Their straightforward operation typically requires minimal training, making them particularly suitable for field applications or point-of-care diagnostics. Furthermore, these sensors hold significant value in clinical diagnosis, where rapid and straightforward detection is prioritized over detailed quantitative analysis. Thus, the proposed strategy, with its potential for low-cost production and rapid results, underscores its relevance in the context of public health.

## 2 Experimental Section

### 2.1 Chemicals

Potassium hexacyanoferrate(II) (Lafan, 98.5%), iron(III) chloride (Synth, 97%), sodium chloride (Dinâmica, 99%), and cetyltrimethylammonium bromide (CTAB, Sigma-Aldrich, 98%) were used to prepare insoluble Prussian blue. Quantitative filter paper (weight: 85 g m^–2^; porosity: 7.50 μm) from Unifil was used as the e-PAD substrate. Phosphate buffer (potassium phosphate monobasic, Exodo, 99%; sodium phosphate dibasic, Vetec, 99%; and potassium chlorite, Exodo, 99%) and Tween 20 (Exodo, 98%) were used for paper disc cleaning. 1-Ethyl-3-(3-(dimethylamino)propyl)carbodiimide (EDC, 99%) and *N*-hydroxysuccinimide (NHS, 98%) from Sigma-Aldrich were used for acidic site activation and antibody immobilization. *Leishmania amazonensis* antibodies and peptide antigens were purified by the Bioprocess Engineering and Biotechnology Graduate Program (Federal University of Paraná, Brazil). Bovine serum albumin (BSA, 99%) from Sigma-Aldrich was used for the blockage of the remaining sites. Hydrogen peroxide (Exodo, 35%) was used as an electrochemical probe. Phosphate-buffered saline (0.10 mol L^–1^ PBS, pH 7.4) was used as the supporting electrolyte. All chemicals used were of analytical grade. Solutions were prepared using ultrapure water (>18 MΩ·cm) obtained from a Milli-Q Plus system (Merck Millipore, USA).

### 2.2 e-PAD Preparation and Modification

**Figure 1:**
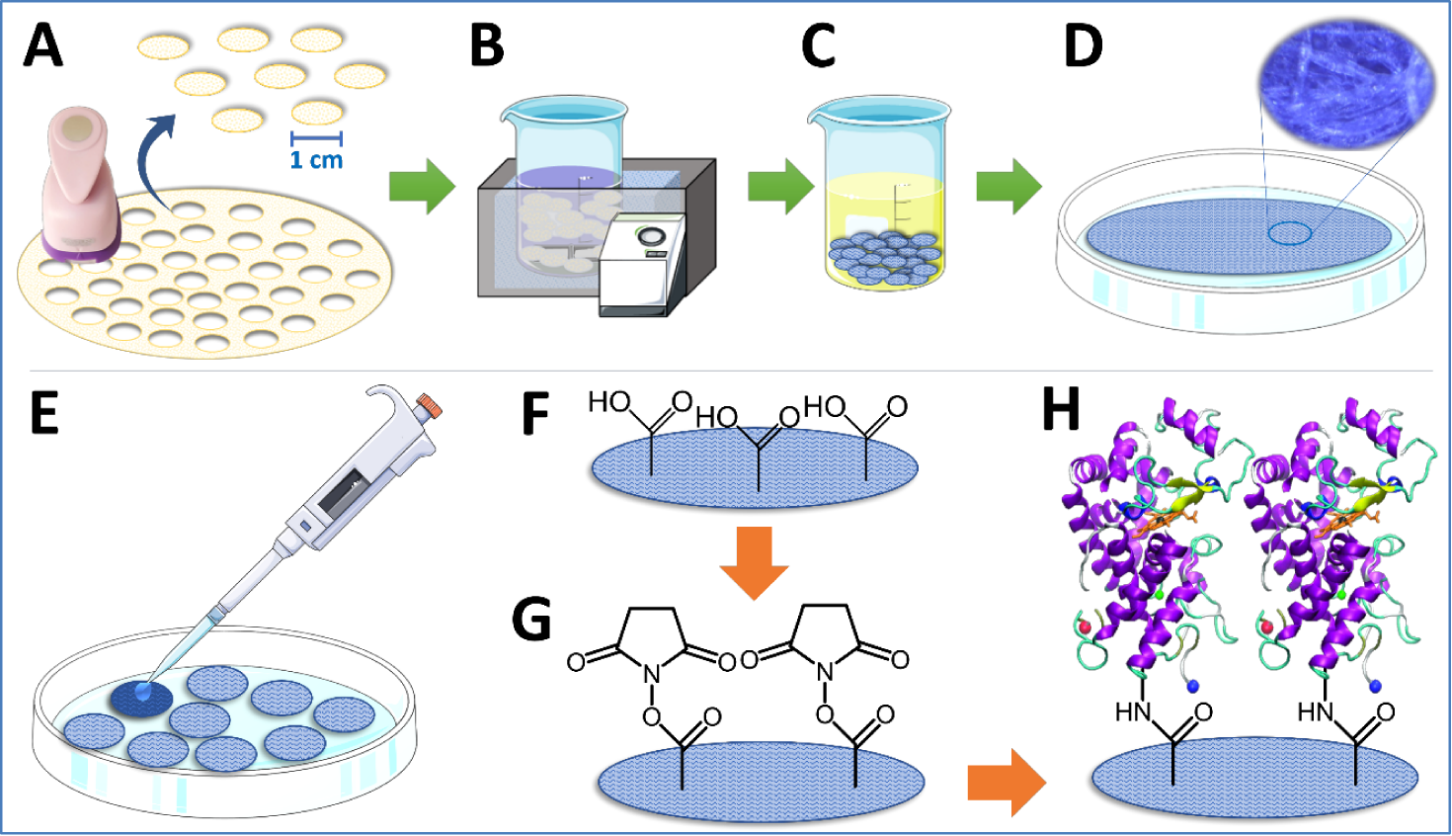
Scheme of paper preparation and modification with Prussian blue (A,D) and modification steps for the construction of the e-PAD immobilized with HRP for hydrogen peroxide detection (E–H).

Paper discs were used for two distinct and complementary functions. First, the discs served as a support for the immobilization of Prussian blue, which acts as an electrocatalyst, enhancing the electrochemical signal. Second, they provided a novel platform for antibody attachment using a method that improves the orientation and stability of the antibodies to ensure effective target interaction. This work introduces a sandwich-type immunosensor architecture using 1 cm diameter paper discs, which were cut with a paper punch, as shown in Figure 1A. The discs were used as the substrate of the immunoassay and were modified by immersion in a Prussian blue solution and remained in an ultrasonic bath (Branson CPX 1800-E, 70 W, frequency of 40 kHz) for 2 h (Figure 1B). The Prussian blue solution was composed of 10 mmol L^–1^ K_4_[Fe(CN)_6_], 20 mmol L^–1^ FeCl_3_·7H_2_O, 0.10 mol L^–1^ NaCl, and 1.0 μmol L^–1^ cetyltrimethylammonium bromide (CTAB). After that, the paper discs were cleaned by immersion in 1.0 μmol L^–1^ NaCl and 0.05% Tween 20 solution for 20 min and again in 1.0 μmol L^–1^ NaCl solution for 20 min (Figure 1C). After the cleaning step, the paper discs impregnated with Prussian blue were dried at 60 °C for 12 h and were ready for the next steps (Figure 1D). Prior to the immobilization of antibodies against *Leishmania*, horseradish peroxidase (HRP) was anchored on paper to evaluate the detection mode based on Prussian blue/Prussian white and hydrogen peroxide. The modifications were performed on the paper discs by drop-casting an aliquot of 40 μL of each modifier (Figure 1E). The activation of the acidic sites was carried out by the reaction with 10 mmol L^–1^ EDC and 40 mmol L^–1^ NHS in PBS for 1 h (Figure 1F,G). After that, the immobilization of the HRP enzyme was promoted by the covalent interaction with amine groups present on the paper substrate (Figure 1H). Subsequently, *Leishmania* antibodies were anchored on the paper following a similar experimental procedure to construct an immunosensor.

### 2.3 Construction of the e-PAD Immunoassay for *Leishmania* Detection

**Figure 2:**
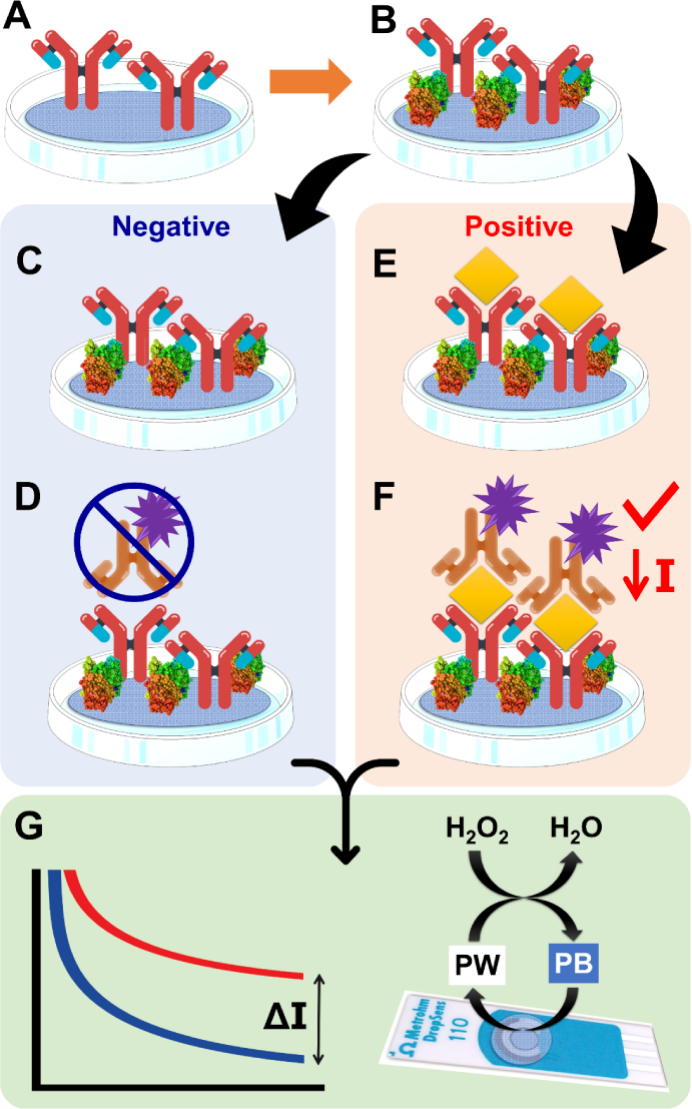
Scheme of modification steps for the construction of the e-PAD immunoassay for the detection of *Leishmania*: anti-*L. amazonensis* antibody immobilization (A), BSA blockage (B), negative (C) and positive control (D), and HRP-conjugated secondary antibody immobilization (E, F). Detection signals obtained for negative and positive samples, according to the representative reaction in the presence of Prussian white (PW) (G).

The proposed electrochemical paper-based immunoassay device (e-PAD) follows a methodology similar to the ELISA kit based on a sandwich-type assembly, using two biological elements, one for capture and one for detection, and a target sandwiched between them.^24^ The modification steps were carried out by the activation of the support and immobilization of capture antibodies, blocking of nonspecific sites, target interaction, HRP-conjugated antibody immobilization, and amperometric response recording (Figure 2). In this case, the surface of the paper was activated using the EDC/NHS pair at 37 °C for 1 h, allowing the formation of carboxylic groups on the paper surface. The capture antibody immobilization was carried out by drop-casting different concentrations of anti-*L. amazonensis* antibodies (40 μL) and heating at 37 °C for 1 h (Figure 2A). After the incubation time, a solution of 1.0 mg mL^–1^ BSA (bovine serum albumin) was used to block residual NHS sites (Figure 2B).

Afterward, the platform was used for the negative control (Figure 2C) where the absence of the target did not lead to any secondary antibodies being labeled on the electrode surface (Figure 2D). The incubation step was carried out in the presence of the peptide (mimicking the target) for 1 h (Figure 2E), aiming for its specific interaction by covalent binding with the antibody light chain. Peptides have a short chain of amino acids connected by amide bonds; thus, they show both amine and carboxylic groups. The modification is followed by incubation with the detection antibodies conjugated with HRP (Figure 2F), forming the sandwich assembly.

The excess labeled antibody was removed by a cleaning step using PBS, and the wet paper disc was placed on the working electrode surface. The HRP-labeled antibodies allow the identification of the formation of the antigen/antibody complex in the presence of hydrogen peroxide. For this, a 10 mmol L^–1^ hydrogen peroxide solution was added to react with the HRP enzyme for 5 min, leading to a partial consumption of hydrogen peroxide, and then, a 0.10 mol L^–1^ H_2_SO_4_ solution was used to stop the reaction by inactivating the HRP. After that, amperometric measurements were performed, providing the reduction of the response signal in the presence of the target. Thus, the differences in the measured currents are related to the presence or absence of the *Leishmania* target in the sample (Figure 2G). After the measurements, the cleaning procedure consisted of removing the e-PAD from the electrode surface and gently rinsing the electrode with ultrapure water to remove any residual reagents, and a new e-PAD was placed above the electrode.

The e-PADs were morphologically characterized by field emission scanning electron microscopy (FE-SEM) using a Quanta 450 FEG microscope before and after the modifications. The size distribution of the antibodies was measured using ImageJ software using the SEM images obtained after the antibody immobilization. The average size was obtained from the measurement of 150 particles. X-ray diffractometry (XRD) was also performed by using a Shimadzu XRD-6000 diffractometer to analyze the presence of Prussian blue structures on the e-PAD.

Aiming to evaluate the biosensor response, a reduced quadratic (optimal RSM design) factorial design was built using the Design-Expert 13 software against the following experimental conditions: capture antibody, peptide, and detection antibody concentrations, from 100 to 5000 μg mL^–1^ (100, 500, 1000, 2500, and 5000 μg mL^–1^), as shown in Table S1.

### 2.4 Electrochemical Measurements

All electrochemical measurements were performed using the e-PAD immunoassay on a Metrohm screen-printed carbon electrode model 110 (silver RE, and carbon WE and CE), coupled to a PalmSens portable potentiostat managed by PSTrace software. Cyclic voltammetry (CV) measurements were carried out for the characterization of the e-PAD, mainly aimed at the catalytic reduction process of hydrogen peroxide. For the measurement steps of the catalytic process, the multiple pulse amperometry (MPA) technique was performed, applying oxidation and reduction potential cycles in which the first pulse is a potential low enough to promote the reduction of Prussian blue to Prussian white, followed by a pulse to oxidize Prussian blue to Berlin green.

### 2.5 Sample Preparation and Analysis

Positive human serum samples contaminated with *L. amazonensis*, with a PCR test, were used. Patients positively diagnosed with leishmaniasis will receive treatment following the guidelines of the Brazilian Ministry of Health, as outlined in the manual of surveillance and control of visceral leishmaniasis.^25^ The second group was composed of blood serum samples collected from healthy people, negative for visceral leishmaniasis. Blood samples from volunteers were collected by trained professionals at a local hospital. After collection, the biological material was subjected to centrifugation and serum separation. The samples were stored at −20 °C and analyzed by the proposed qualitative electrochemical method and by the PCR test. This study is approved by the Research Ethics Committee of the Federal University of Paraná, Brazil (CEP/SD 428.108.07.10). The selectivity of the e-PAD immunoassay for *Leishmania* was evaluated for the detection of the yellow fever virus as the negative target, following the same experimental conditions.

## 3 Results and Discussion

### 3.1 Morphological Characterization of the Paper-Based Immunoassay and Voltammetric Behavior of the System

**Figure 3:**
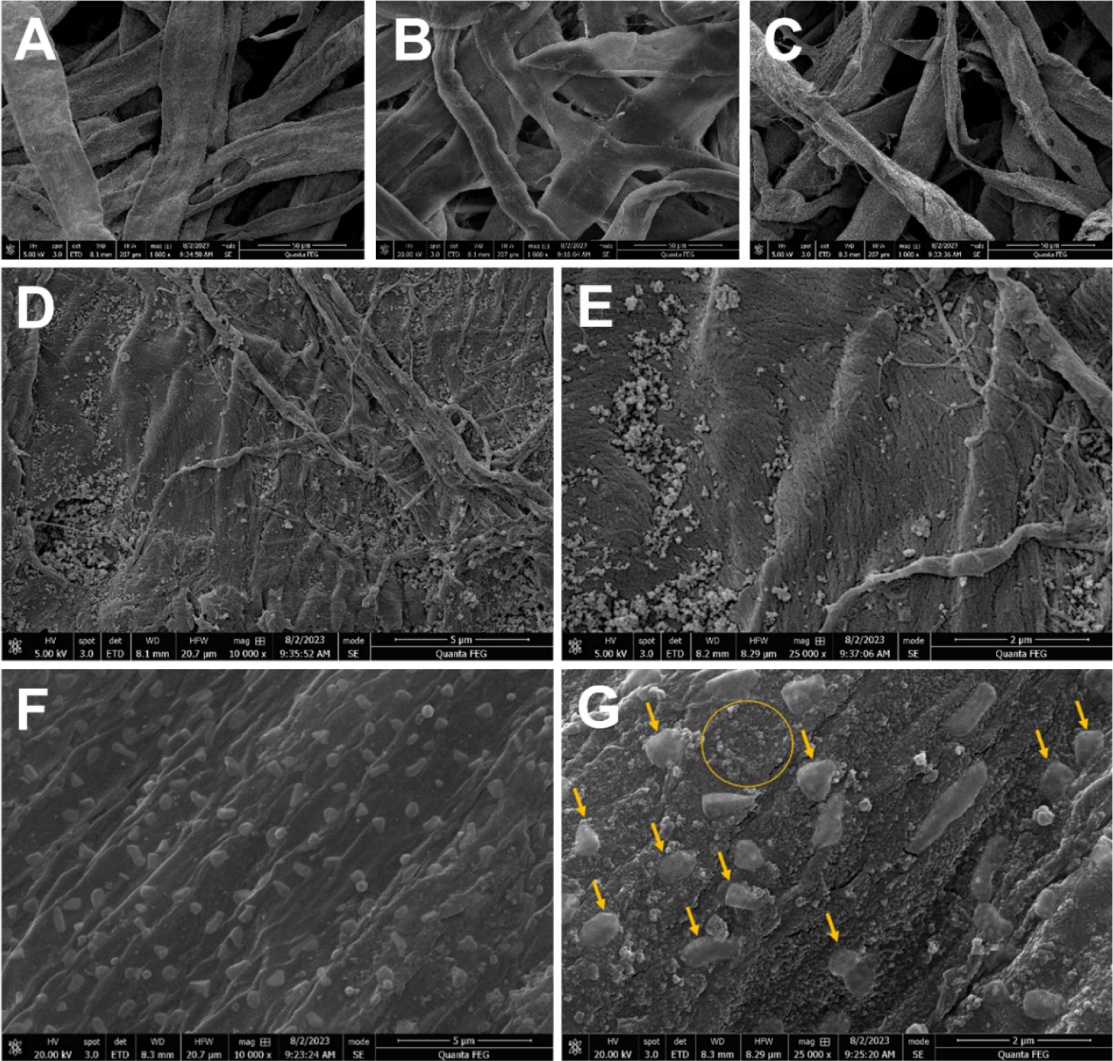
SEM images obtained for the paper substrate before modification (A) and after the impregnation of Prussian blue (B–F) and the immobilization of the capture antibody (Ab1) (C–G) with different magnifications. Highlighted: Prussian blue nanostructures (yellow cycle) and immobilized antibodies (yellow arrows).

The morphology of the paper surface was investigated by using SEM measurements recorded after each step of the bioassay assembly. SEM representative images of the paper substrate as well as the e-PAD immunoassay constructed by immobilizing the redox mediator Prussian blue and the recognition site are shown in Figure 3A–G. First, the paper revealed a homogeneous structure where the fibers can be observed (Figure 3A–C). The presence of Prussian blue nanostructures (<200 nm) can be observed on the fibers of the paper substrate, suggesting that this modification was effective (Figure 3D,F). In the same way, capture antibodies were successfully stabilized and immobilized on the paper substrate (Figure 3E,G) showing a homogeneous distribution on the entire surface. As highlighted in Figure 3G, the yellow cycle indicates a region containing the presence of Prussian blue nanostructures, and the arrows indicate the antibody particles. Antibody clusters showed an average size of 505 ± 171 nm, as shown in Figure S1. This SEM image illustrates the successful immobilization of both the irregular, aggregated antibody clusters and Prussian blue, displaying its characteristic crystalline cubic shape.

The X-ray diffractometry (XRD) technique was used to corroborate the analysis of the paper incorporated with Prussian blue. Figure S2 shows the spectra obtained, in which diffraction peaks observed at 22.9°, 16.9°, and 34.7° correspond to the 220, 200, and 400 crystallographic planes, respectively. These peaks indicate a face-centered cubic (FCC) structure. The 220 plane observed at 22.9° reflects a specific atomic arrangement within the cubic lattice, while the 200 plane at 16.9° shows a different layer spacing that supports the FCC structure. The 400 plane at 34.7° confirms higher-order reflections, consistent with the periodic atomic arrangement found in Prussian blue.

For the electrochemical measurements, the e-PAD is placed on the screen-printed electrode, as shown in Figure S3, and a drop of solution of 50 μL is used to perform the measurements. The electrochemical behavior of the paper containing immobilized Prussian blue (PB) was evaluated through cyclic voltammetric measurements to demonstrate the effectiveness of the formed complex compound (Figure S4). The measurements were performed in a solution containing 0.10 mol L^–1^ PBS and 1.0 mol L^–1^ H_2_SO_4_. Typical redox processes related to the Prussian blue were observed, with the first redox couple, PB/PW (Prussian blue/Prussian white), occurring at +0.41 and +0.25 V (vs Ag|AgCl), and the second couple, PB/BG (Prussian blue/Berlin green), occurring at +1.37 and +0.62 V (vs Ag|AgCl). Successive voltammetric cycles showed an increase in the intensity of the anodic current peaks related to the oxidation of Prussian white to Prussian blue, as highlighted in the inset. No significant current variation was observed after 5 cycles, indicating the stabilization of the formed complex on the substrate.

**Figure 4:**
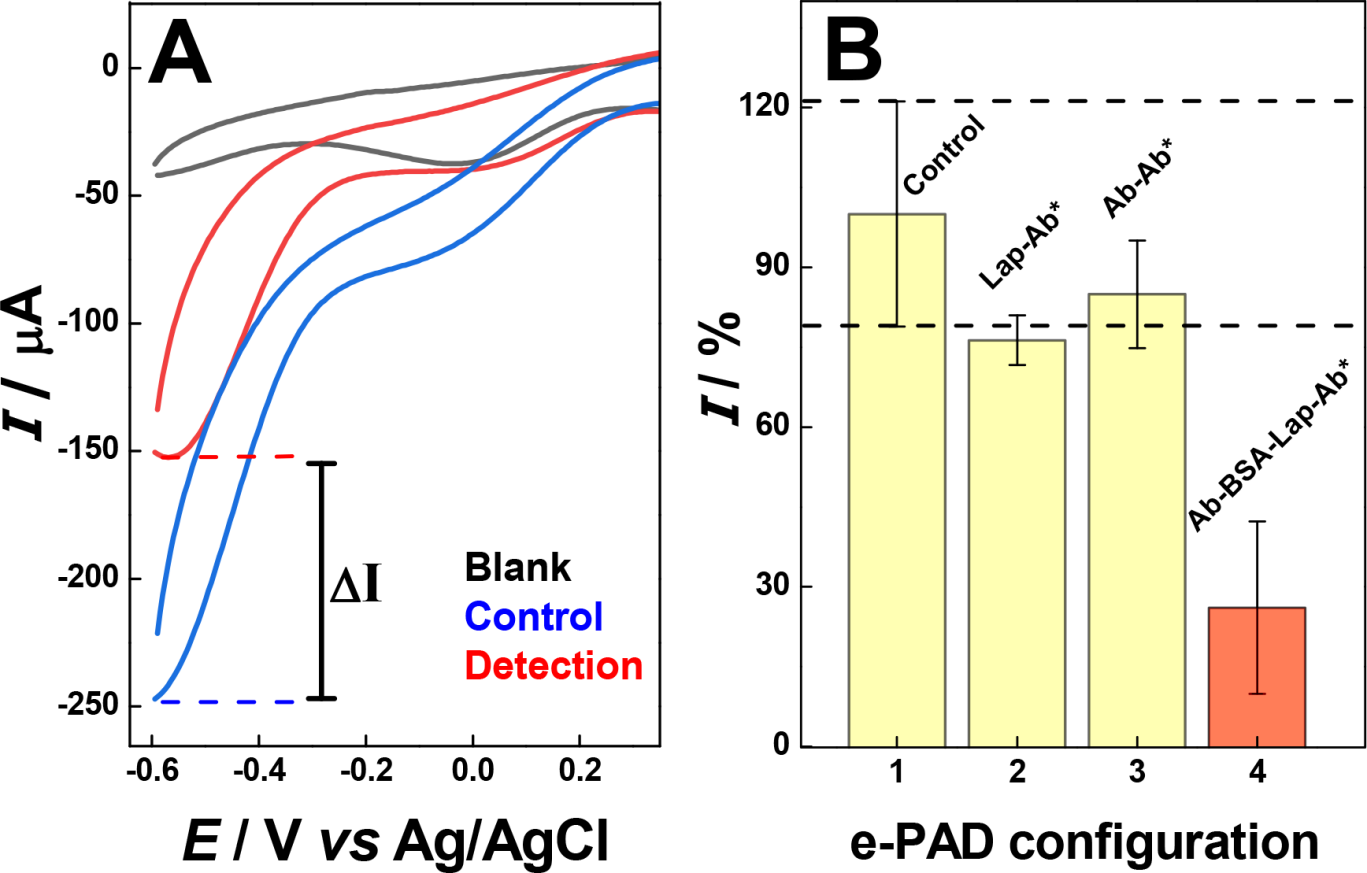
(A) Cyclic voltammograms obtained for different paper-based immunoassay supports in the absence of H_2_O_2_ (blank), in the presence of 0.01 vol % H_2_O_2_ after (control), and before incubation in the presence of target (detection). Supporting electrolyte: 0.10 mol L^–1^ PBS + 1.0 mol L^–1^ H_2_SO_4_. Scan rate: 50 mV s^–1^. (B) Relative current intensity obtained by chronoamperometry measurements performed for the e-PAD sandwich-type immunosensor construction.

The evaluation of the bioassay was performed on paper containing both the recognition site and immobilized PB in an acid solution (Figure 4A) in the absence (blank) and presence of H_2_O_2_ before (control) and after incubation in the presence of the target (detection). In this condition, PW chemically reacts with hydrogen peroxide (H_2_O_2_), promoting its reduction (H_2_O_2_ + 2e^–^ → 2OH^–^) and yielding PB.^26,27^ The chemically formed PB can undergo reduction to PW on the electrode surface, resulting in an enhancement of the cathodic current related to the PB/PW electrochemical process (Figure 4A, control) compared to the absence of peroxide (Figure 4A, blank). When the platform was subjected to incubation in the presence of the target, followed by incubation with secondary antibodies labeled with HRP, the presence of the enzyme led to the partial decomposition of hydrogen peroxide. This variation affects the catalytic cycle related to PW/PB, resulting in a decrease in the cathodic current (Figure 4A, detection). Differences in the cathodic currents were attributed to the presence or absence of the target in the sample. The relative current for positive and negative samples was calculated as a percentage (%I) relative to the control value, which was standardized to 100%.

Aiming to promote a better electrochemical response for the reduction of H_2_O_2_ by chronoamperometry, the influence of some experimental parameters was studied and optimized. The best conditions were obtained at 0.10 vol % H_2_O_2_, an applied potential of −0.40 V (vs. Ag/AgCl), and H_2_O_2_ reaction time of 5 min (Figure S5). For multiple pulse amperometry (MPA), 20 pulses were applied during 200 ms under optimized conditions (Figure S6). The application of an experimental design to determine the optimal conditions for the capture antibody at 100 μg mL^–1^, peptide at 100 μg mL^–1^, and detection antibody fixed at 500 μg mL^–1^ on the e-PAD surface proved to be effective for performing the detection (Figure S7). Also, analysis of variance (ANOVA) showed that the model is significant, with an F value of the model of 4.43 and a p-value less than 0.05, as shown in Table S2. Among all the statistical parameters used in mathematical modeling, the adequate precision is of great criterion, and a value of 8.49 was obtained (see Table S3). Therefore, the method is considered satisfactory since the value statistically used to consider that the method is carried out with adequate precision is above 4.^28^

After the optimization of experimental conditions, the e-PAD immunosensor was evaluated after different assembly configurations, as shown in Figure 4B: **1.** capture antibodies and BSA (control); **2.***L. amazonensis* peptide and HRP-conjugated antibodies (Lap-Ab*); **3.** capture antibodies and HRP-conjugated antibodies (Ab-Ab*); and **4.** capture antibodies, *L. amazonensis* peptide, and HRP-conjugated antibodies modifications (Ab-BSA-Lap-Ab*). The first configuration (1) is described as a basal signal that serves as a baseline for comparison. Configurations 2 and 3 exhibit cathodic currents very close to the blank, suggesting that they have a similar content of H_2_O_2_ on the electrode surface. For complete sandwich configuration 4 (Ab-BSA-Lap-Ab*) used for the detection of the peptide, the presence of antibodies labeled with HRP leads to a significant variation in the recorded current due to enzymatic peroxide consumption. This result indicates that the immunosensor can effectively detect the presence of *Leishmania* and distinguish it from the control response signal (blank) with a 95% confidence interval, as verified by Student’s *t*-test. The performance of the immunosensor is directly dependent on a series of modification steps. In these steps, capture antibodies interact specifically with *Leishmania* peptides. Subsequently, these peptides were recognized by detection of antibodies labeled with the HRP enzyme. This interaction forms a sandwich-type immunosensor.

The positive detection is achieved indirectly through the specific interaction of the peptide with the sandwich immunosensor. In this approach, the reduction of H_2_O_2_ is catalyzed by the presence of the HRP enzyme and Prussian blue used as the redox mediator.^29^ Thus, the electrochemical detection of *Leishmania* was carried out by amperometry using Prussian blue as the redox mediator in the presence of hydrogen peroxide (H_2_O_2_). The concentration of H_2_O_2_ can be directly related to the reaction and detection of the Leishmania peptide. Figure S8 compares the currents obtained by amperometry for the blank values (control) and the detection of *Leishmania* peptide using the e-PAD sandwich immunosensor at different H_2_O_2_ concentrations (0.005–0.1 vol %). The optimal experimental condition for detecting the peptide was verified at 0.01 vol % H_2_O_2_, which was then used for further studies.

The reproducibility of the bioassay-paper platform plays an important role and can significantly affect test performance. Thus, six different constructed immunosensors were evaluated, showing excellent reproducibility (RSD = 5.12%, *n* = 6) (Figure S9). Also, the stability of the papers was evaluated before and after the previous treatments and after electrochemical measurements, in which no significant variations were noted after treatments and measurements, as shown in the pictures of the papers (Figure S10). After preparation, the paper shows an intact aspect, with no apparent defects, demonstrating that it maintains its integrity even after the preparation treatments. After use, the paper presents a slight color variation, which may be due to the formation of Prussian white or other subproducts. Thus, it is possible to highlight that the proposed e-PAD bioassay is a stable, low-cost, and eco-friendly alternative for sensor modification. By addressing these features through systematic experimental approaches and quality assurance protocols, it is possible to enhance the reliability of electrochemical sensor measurements.

### 3.2 Interference and Human Sample Analysis

**Figure 5:**
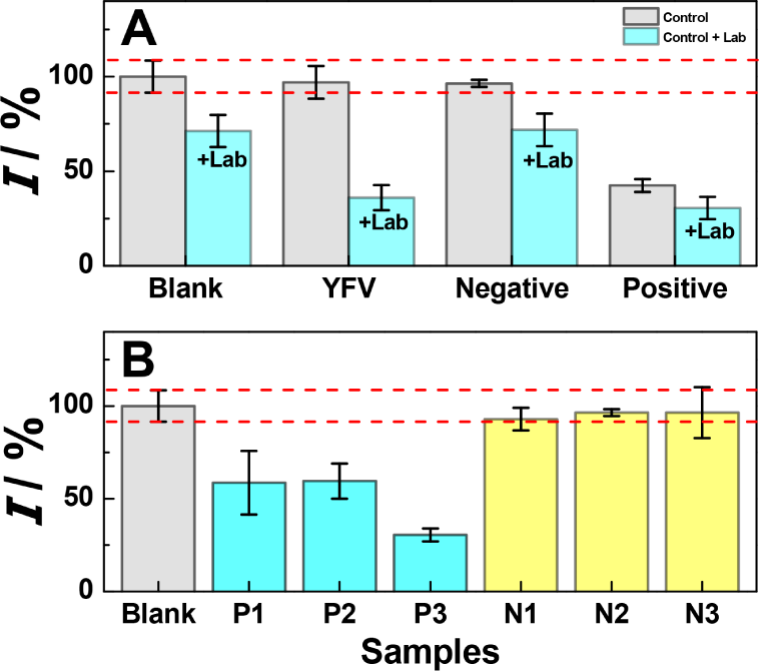
(A) Selectivity analysis performed for *L. amazonensis* peptide detection against yellow fever virus (YFV), negative samples, and positive samples as interferents in the absence (control) and presence of the target (control + Lab). (B) Sample analysis performed for *Leishmania* negative and positive human blood serum samples (*n* = 3).

The selectivity of the immunosensor was investigated against yellow fever virus (YFV) as a potential interferent (Figure 5A). The representative amperometric responses used to prepare Figure 5 are provided in Figure S11. No significant difference in the signal was observed in comparison with the blank and after incubation in the presence of the concomitant, demonstrating that the proposed immunosensor can be considered selective toward *Leishmania* peptide. Similar studies were carried out using negative and positive samples. The experimental results demonstrated that the negative sample initially exhibited a response close to the blank, but after the addition of Lab, it yielded a positive result. The positive sample, which already showed a pronounced response, further intensified after Lab addition. For the qualitative application of the proposed e-PAD immunosensor, human blood serum of three volunteers diagnosed with *Leishmania* and three control samples were tested (Figure 5B). When the e-PAD was tested in three infected volunteers, a marked response in comparison to the blank conditions was demonstrated. Statistical analysis further confirmed the significance of these findings, revealing that the calculated t-Student value exceeded the critical t-value at a significance level of α = 0.05 (Table S4). This demonstrates that the observed values differ significantly from those of the control. In contrast, no significant variance was observed for the test conducted on the control group compared with the sensor blank. These results demonstrate that the proposed e-PAD immunosensor can be effectively applied for detecting infections, while its response remains negligible in noninfected individuals, emphasizing its potential utility as a diagnostic tool in clinical settings.

Thus, as observed in Table 1, electrochemical-based bioassays are successfully applied for the detection of different *Leishmania* targets (i.e., antibodies, DNA, and peptides), showing remarkable distinguishability and sensitivity between positive and negative samples and other diseases.

**Table 1:** Comparison between Biosensors Applied for the Detection of *Leishmania*

Biosensor	Technique	Target	Sample analysis	Selectivity analysis	ref.
AuNP-SPE	CV	Visceral leishmaniasis antibody	Visceral serum	Chagas disease	(5)
GO- or GQD-SPE	DPV	*Leishmania amazonensis* antibody	Human and canine blood serum	*Mycobacterium leprae* and tuberculosis	(18)
GNL/Au	DPV	*Leishmania major* DNA	Human biopsy specimens	Noncomplementary and *Leishmania tropica* DNA	(30)
PAD PGE	EIS	American tegumentary leishmaniasis antibody	Human blood serum	a	(31)
Cu-(NH_2_–BDC) MOF AuSPE	EIS	*Leishmania major* gp63 antibody	*Leishmania* parasite crude antigen and rabbit serum	Anti-VEGF, anti-AFP, and anti-IgG antibodies	(32)
e-PAD SPCE	*MPA*	*Leishmania amazonensis* peptide	Human blood serum	Yellow fever virus	This work

a Data not informed. AuNP-SPE: screen-printed carbon electrode modified with gold nanoparticles; Cu-(NH_2_–BDC) MOF AuSPE: Gold screen-printed carbon electrode modified with the synthesized Cu-(NH_2_–BDC) metal–organic framework; CV: cyclic voltammetry; DPV: differential pulse voltammetry; EIS: electrochemical impedance spectroscopy; e-PAD SPCE: electrochemical paper-based analytical device on a screen-printed carbon electrode; GO- or GQD-SPE: screen-printed carbon electrode modified with graphene oxide or graphene quantum dots; GNL/Au: gold nanoleaves-coated gold disc electrode; MPA: multiple pulse amperometry; PAD PGE: paper-based electrochemical platform with pencil graphite electrodes.

## 4 Conclusion

A novel electrochemical paper-based analytical device (e-PAD) was demonstrated for the qualitative diagnosis of leishmaniasis. The proposed immunoassay provides a portable, low-cost, and rapid solution for detecting leishmaniasis antigens, making it highly suitable for point-of-care diagnostics. The e-PAD was constructed by immobilizing Prussian blue and antibodies specific to Amazonian leishmaniasis on a paper substrate, which was then combined with unmodified, screen-printed electrodes to assemble the electrochemical immunoassay. Notably, the modification performed on paper discs allows the use of different electrode shapes, showcasing the versatility of this approach. The e-PAD was successfully applied to positive and negative samples, exhibiting good reproducibility and selectivity. This work highlights the potential of e-PADs as rapid, reliable, and accessible diagnostic tools for neglected diseases such as leishmaniasis, addressing critical healthcare needs in underserved populations.

## Supporting Information Available

Factorial design, antibody size distribution, X-ray diffraction (XRD) spectra, e-PAD bioassay assembly, electrochemical behavior of PB immobilized on paper support, parameter optimization for H_2_O_2_ detection, parameter optimization of the e-PAD immunosensor, multivariate optimization (factorial design), effect of hydrogen peroxide, reproducibility of the e-PAD bioassay, interference evaluation, and sample analysis (PDF)

## References

[ref1] Afonso R. C.; Yien R. M. K.; de Siqueira L. B. D. O.; Simas N. K.; Matos A. P. S.; Ricci-Júnior E. Promising natural products for the treatment of cutaneous leishmaniasis: A review of in vitro and in vivo studies. Exp. Parasitol. 2023, 251, 108554 10.1016/j.exppara.2023.108554. 37268108

[ref2] Mokni M. Leishmanioses cutanéesCutaneous leishmaniasis. Ann. Dermatol. Venereol. 2019, 146 (3), 232–246. 10.1016/j.annder.2019.02.002. 30879803

[ref3] Vega D. M.; Di Meglio M.; Alonso S. D. V.; Alvira F.; Montanari J. Nanomaterials for diagnosis, treatment, and prevention of human cutaneous leishmaniasis: A review. OpenNano 2023, 12, 100158 10.1016/j.onano.2023.100158.

[ref4] Santos T. N. V.; Silva E. R.; Teles J. S.; Cunha Í. T. F.; Prado M. C. M. N.; Santos-Júnior R. B.; Santos G. R. S.; Moura T. R.; Lima S. V. M. A.; Santos A. D.; et al. CARACTERIZAÇÃO DOS ÓBITOS POR LEISHMANIOSE VISCERAL NO BRASIL (2012-2019). Braz. J. Infect. Dis. 2023, 27, 103519 10.1016/j.bjid.2023.103519.

[ref5] Martins B. R.; Barbosa Y. O.; Andrade C. M. R.; Pereira L. Q.; Simão G. F.; Oliveira C. J.; Correia D.; Oliveira-Jr R. T. S.; Silva M. V.; Silva A. C. A.; et al. Development of an Electrochemical Immunosensor for Specific Detection of Visceral Leishmaniasis Using Gold-Modified Screen-Printed Carbon Electrodes. Biosensors 2020, 10, 81 10.3390/bios10080081. 32717832 PMC7460044

[ref6] Thakur P. S.; Sankar M. Nanobiosensors for biomedical, environmental, and food monitoring applications. Mater. Lett. 2022, 311, 131540 10.1016/j.matlet.2021.131540.

[ref7] Felix F. S.; Angnes L. Electrochemical immunosensors – A powerful tool for analytical applications. Biosens. Bioelectron. 2018, 102, 470–478. 10.1016/j.bios.2017.11.029. 29182930

[ref8] Biasotto G.; Costa J. P. C.; Costa P. I.; Zaghete M. A. ZnO nanorods-gold nanoparticle-based biosensor for detecting hepatitis C. Appl. Phys. A: Mater. Sci. Process. 2019, 125, 821 10.1007/s00339-019-3128-1.

[ref9] Bong J.-H.; Kim H.-R.; Jung J.; Park J.-H.; Sung J. S.; Lee C. K.; Choi K.-H.; Shin S.-S.; Kang M.-J.; Kim H. O.; et al. Switching-peptides for one-step immunoassay and its application to the diagnosis of human hepatitis B. J.-C.Biosens. Bioelectron. 2021, 178, 112996 10.1016/j.bios.2021.112996. 33524706

[ref10] Joshi S. R.; Sharma A.; Kim G.-H.; Jang J. Low cost synthesis of reduced graphene oxide using biopolymer for influenza virus sensor. Mater. Sci. Eng 2020, 108, 110465 10.1016/j.msec.2019.110465. 31924022

[ref11] Mostafa I. M.; Tian Y.; Anjum S.; Hanif S.; Hosseini M.; Lou B.; Xu G. Comprehensive review on the electrochemical biosensors of different breast cancer biomarkers. Sens. Actuators, B 2022, 365, 131944 10.1016/j.snb.2022.131944.

[ref12] Nawaz M. H.; Hayat A.; Catanante G.; Latif U.; Marty J. L. Development of a portable and disposable NS1 based electrochemical immunosensor for early diagnosis of dengue virus. Anal. Chim. Acta 2018, 1026, 1 10.1016/j.aca.2018.04.032. 29852984

[ref13] Cabral-Miranda G.; Cardoso A. R.; Ferreira L. C. S.; Sales M. G. F.; Bachmann M. F. Biosensor-based selective detection of Zika virus specific antibodies in infected individuals. Biosens. Bioelectron. 2018, 113, 101 10.1016/j.bios.2018.04.058. 29751200

[ref14] Cerrutti B. M.; Moraes M. L.; Pulcinelli S. H.; Santilli C. V. Lignin as immobilization matrix for HIV p17 peptide used in immunosensing. Biosens. Bioelectron. 2015, 71, 420 10.1016/j.bios.2015.04.054. 25950938

[ref15] Gogola J. L.; Martins G.; Gevaerd A.; Blanes L.; Cardoso J.; Marchini F. K.; Banks C. E.; Bergamini M. F.; Marcolino-Junior L. H. Label-free aptasensor for p24-HIV protein detection based on graphene quantum dots as an electrochemical signal amplifier. Anal. Chim. Acta 2021, 1166, 338548 10.1016/j.aca.2021.338548. 34022998

[ref16] Soto D.; Orozco J. Peptide-based simple detection of SARS-CoV-2 with electrochemical readout. Anal. Chim. Acta 2022, 1205, 339739 10.1016/j.aca.2022.339739. 35414399 PMC8935448

[ref17] Martins G.; Galeski H. R.; Andrade G. A.; Valenga M. G. P.; Ramos M. K.; Zarbin A. J. G.; Janegitz B. C.; Müller-Santos M.; de Souza E. M.; Marcolino-Junior L. H.; Bergamini M. F. One-step selective layer assemble: A versatile approach for the development of a SARS-CoV-2 electrochemical immunosensor. Anal. Chim. Acta 2023, 1278, 341726 10.1016/j.aca.2023.341726. 37709467

[ref18] Braz B. A.; Hospinal-Santiani M.; Martins G.; Beirão B. C. B.; Bergamini M. F.; Marcolino-Junior L. H.; Soccol C. R.; Thomaz-Soccol V. Disposable electrochemical platform based on solid-binding peptides and carbon nanomaterials: an alternative device for leishmaniasis detection. Microchim. Acta 2023, 190, 321 10.1007/s00604-023-05891-z. 37491620

[ref19] Ma J.; Jiang G.; Ma Q.; Du M.; Wang H.; Wu J.; Wang C.; Xie X.; Li T.; Chen S.; Zhang L.; et al. Portable immunosensor directly and rapidly detects Mycobacterium tuberculosis in sputum. Anal. Methods 2022, 14 (4), 438–448. 10.1039/D1AY01561C. 35022623

[ref20] Kasetsirikul S.; Shiddiky M. J.; Nguyen N.-T. Challenges and perspectives in the development of paper-based lateral flow assays. Microfluid. Nanofluid. 2020, 24, 17 10.1007/s10404-020-2321-z.

[ref21] Li Z.; Liu H.; He X.; Xu F.; Li F. Pen-on-paper strategies for point-of-care testing of human health. TrAC, Trends Anal. Chem. 2018, 108, 50–64. 10.1016/j.trac.2018.08.010.

[ref22] Seddaoui N.; Colozza N.; Gullo L.; Arduini F. Paper as smart support for bioreceptor immobilization in electrochemical paper-based devices. Int. J. Biol. Macromol 2023, 253, 127409 10.1016/j.ijbiomac.2023.127409. 37848114

[ref23] Alahmad W.; Cetinkaya A.; Kaya S. I.; Varanusupakul P.; Ozkan S. A. Electrochemical paper-based analytical devices for environmental analysis: Current trends and perspectives. Trends Environ. Anal. Chem. 2023, 40, e00220 10.1016/j.teac.2023.e00220.

[ref24] Kalinke C.; Oliveira P. R.; Bonacin J. A.; Janegitz B. C.. Chapter 3 - Biosensors: an introduction Biosensors in Precision Medicine; 1st, ed Elsevier: Amsterdam, 2024 pp. 61–104.

[ref25] Cerqueira L.; Alves L.; Pamplona M.; Bastos F. Manual of surveillance and control of visceral leishmaniasis, 1st ed.; Brazilian Ministry of Health: Brasilia, 2014; pp. 1–120.

[ref26] Karyakin A. A.; Karyakina E. E.; Gorton L. On the mechanism of H_2_O_2_ reduction at Prussian Blue modified electrodes. Electrochem. Commun. 1999, 1, 78–82. 10.1016/S1388-2481(99)00010-7.

[ref27] Noël J. M.; Médard J.; Combellas C.; Kanoufi F. Prussian Blue Degradation during Hydrogen Peroxide Reduction: A Scanning Electrochemical Microscopy Study on the Role of the Hydroxide Ion and Hydroxyl Radical. ChemElectrochem 2016, 3, 1178–1184. 10.1002/celc.201600196.

[ref28] Dritsa V.; Rigas F.; Doulia D.; Avramides E. J.; Hatzianestis I. Optimization of Culture Conditions for the Biodegradation of Lindane by the Polypore Fungus Ganoderma australe. Water, Air, Soil Pollut. 2009, 204, 19–27. 10.1007/s11270-009-0022-z.

[ref29] Fedalto L.; Oliveira P. R.; Agustini D.; Kalinke C.; Banks C. E.; Bergamini M. F.; Marcolino-Junior L. H. Novel and highly stable strategy for the development of microfluidic enzymatic assays based on the immobilization of horseradish peroxidase (HRP) into cotton threads. Talanta 2023, 252, 123889 10.1016/j.talanta.2022.123889. 36070669

[ref30] Moradi M.; Sattarahmady N.; Rahi A.; Hatam G. R.; Sorkhabadi S. M. R.; Heli H. A label-free, PCR-free and signal-on electrochemical DNA biosensor for Leishmania major based on gold nanoleaves. Talanta 2016, 161, 48–53. 10.1016/j.talanta.2016.08.030. 27769435

[ref31] Barraza D. E.; Nanni P. I.; Bracamonte M. E.; Chaile R. E.; Goy C. B.; Acuña L.; Marco J. D.; Madrid R. E. Simple and promising paper-based electrochemical platform for serological detection of American tegumentary leishmaniasis. Mem. Inst. Oswaldo Cruz. 2024, 119, e230149 10.1590/0074-02760230149. 38359306 PMC10868376

[ref32] Perk B.; Büyüksünetçi Y. T.; Bouzaien S. B.; Diouani M. F.; Anik Ü. Microchem. J. 2023, 192, 108958 10.1016/j.microc.2023.108958.

