# Supplementary material for: Electrochemical Paper-Based Analytical Device (e-PAD) Using Immobilized Prussian Blue and Antibodies for the Diagnosis of Leishmania
Source: ACS Omega. 2025 Feb 12;10(7):6593–600. doi: 10.1021/acsomega.4c07455 (PMC11866208; doi:10.1021/acsomega.4c07455)
Supplement: Supplementary file 1 — ao4c07455_si_001.pdf [file ao4c07455_si_001.pdf]

Supplementary Material

Electrochemical Paper-Based Analytical Device (e-PAD) Using Immobilized Prussian Blue and Antibodies for the Diagnosis of Leishmania

Maurício Alberto Poletti Papi[a], Cristiane Kalinke[a,b], Carlos R. Soccol[c], Vanete Thomaz Soccol[c], Breno C. B. Beirão[d], Márcio F. Bergamini[a], and Luiz H. Marcolino-Júnior[a*]

SUMMARY

Factorial Design (Table S1)	***1***
Antibodies Size Distribution (Figure S1)	***2***
X-ray diffraction (XRD) spectra (Figure S1)	***2***
e-PAD Bioassay Assembly (Figure S3)	***3***
Electrochemical behavior of PB immobilized on paper support (Figure S4)	***3***
Parameter Optimization for H_2O_2 Detection (Figure S5)	***4***
Parameter Optimization of the e-PAD immunosensor (Figure S6)	***4***
Multivariate Optimization (Factorial Design) (Figure S7, Tables S2 e S3)	***5-6***
Effect of hydrogen peroxide (Figure S8)	***6***
Reproducibility of the e-PAD Bioassay (Figure S9 and S10)	***7***
Interference evaluation (Figure S11)	***8***
Sample Analysis (Table S4)	***9***

* Corresponding authors:

bergamini@ufpr.br (M.F. Bergamini) luiz1berto@ufpr.br (L. H. Marcolino-Júnior).

Factorial Design

Table S1. Reduced quadratic factorial design adopted for the optimization of capture antibody (Factor 1), peptide (Factor 2), and detection antibody (Factor 3) concentrations aiming the immunosensor construction.

Block	Factor 1 – A [Ab_1] (µg mL^{-1})	Factor 2 – B [Pep] (µg mL^{-1})	Factor 3 – C [Ab_2*] (µg mL^{-1})	Response
**Block 1**	100	2550	2550	432
**Block 1**	800	4300	4300	508
**Block 1**	5000	2550	2550	498
~~**Block 1**~~	~~800~~	~~800~~	~~800~~	~~652~~
~~**Block 1**~~	~~4300~~	~~4300~~	~~800~~	~~397~~
~~**Block 1**~~	~~4300~~	~~4300~~	~~800~~	~~316~~
**Block 1**	4300	800	4300	364
~~**Block 1**~~	~~800~~	~~800~~	~~800~~	~~377~~
**Block 1**	100	2550	2550	492
~~**Block 1**~~	~~800~~	~~4300~~	~~4300~~	~~652~~
**Block 1**	4300	800	4300	435
**Block 1**	5000	2550	2550	524
**Block 2**	2550	5000	2550	676
**Block 2**	2550	2550	2550	656
**Block 2**	2550	2550	100	516
**Block 2**	2550	100	2550	517
~~**Block 2**~~	~~2550~~	~~2550~~	~~5000~~	~~383~~
**Block 2**	2550	2550	5000	576
**Block 2**	2550	5000	2550	630
**Block 2**	2550	2550	2550	613
**Block 2**	2550	2550	100	510
**Block 2**	2550	100	2550	492
**Block 3**	2550	2550	2550	681
**Block 3**	2550	2550	2550	587
**Block 3**	2550	2550	2550	540
**Block 3**	2550	2550	2550	560
~~**Block 3**~~	~~2550~~	~~2550~~	~~2550~~	~~447~~
**Block 3**	2550	2550	2550	704
**Block 3**	2550	2550	2550	643

* Strikethrough lines represent the runs not used in this Model.

Antibodies Size Distribution

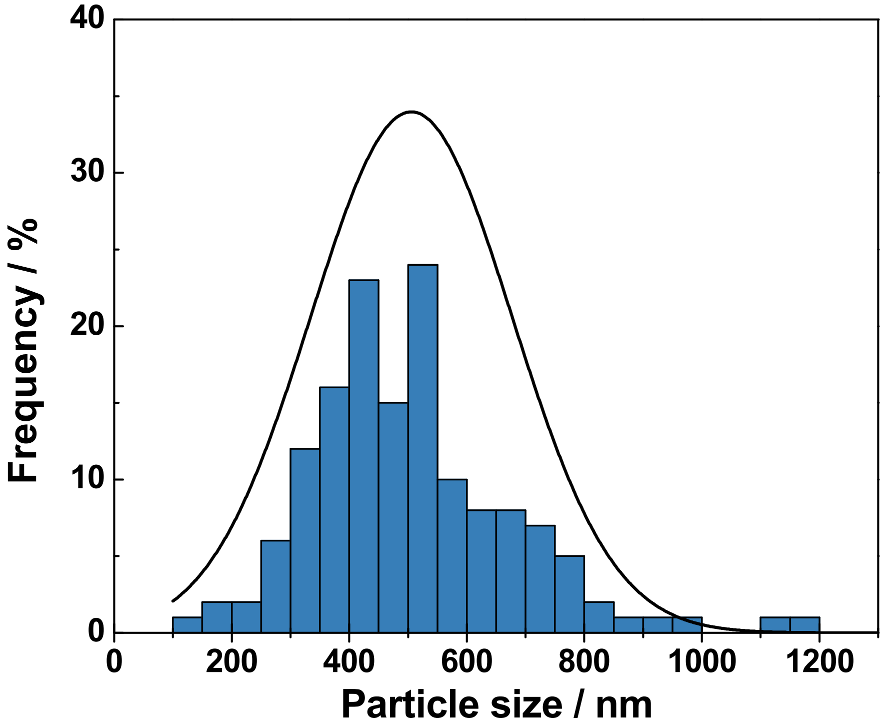

Figure S2. Antibodies particle size distribution with average size of 505 ±171 nm.

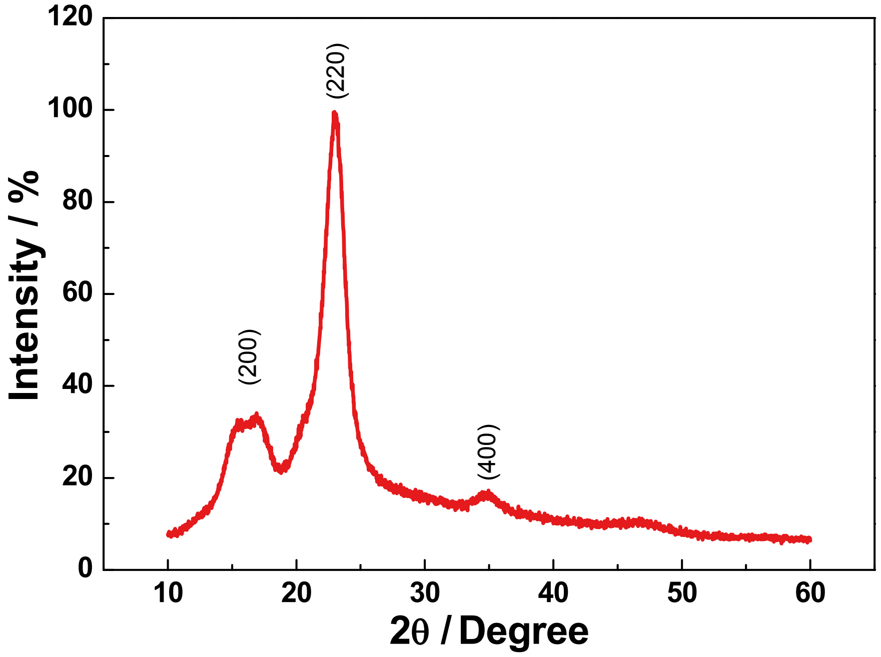

Figure S3. X-ray diffraction (XRD) spectra obtained for the paper incorporated with Prussian blue sample.

e-PAD Bioassay Assembly

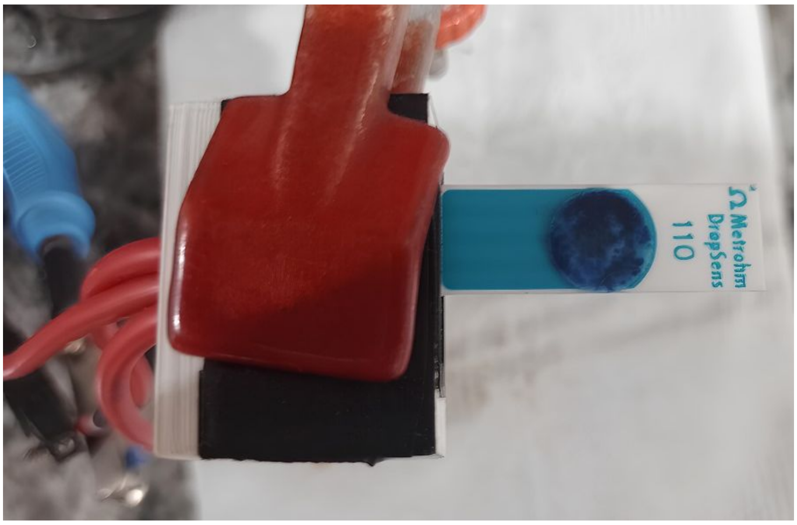

Figure S4. Photograph of the e-PAD on the screen-printed electrode surface for the electrochemical measurements.

Electrochemical behavior of PB immobilized on paper support

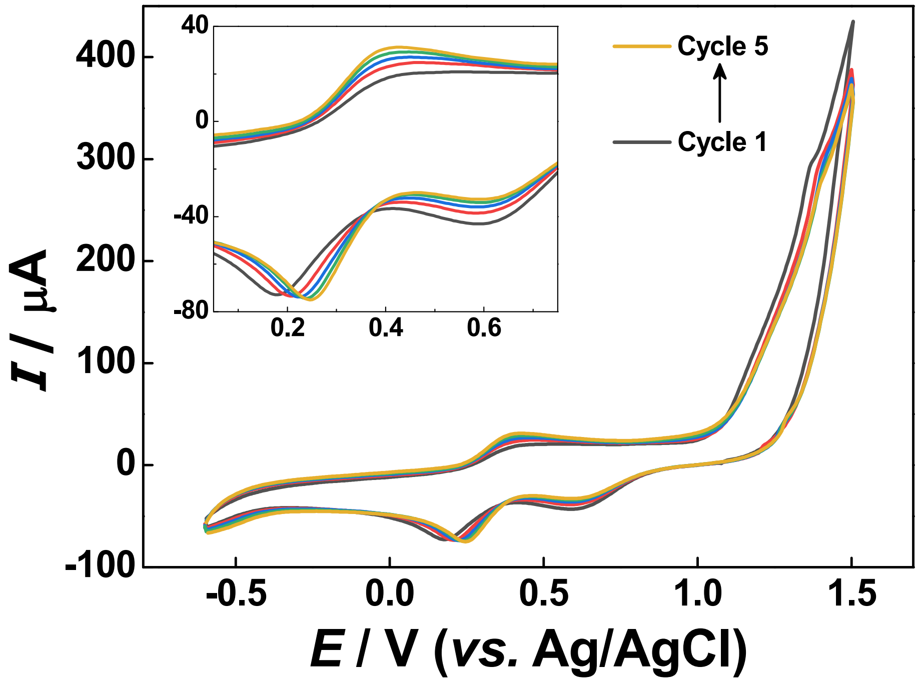

Figure S5. Cyclic voltammograms obtained for the Prussian blue electrochemical behavior at different cycles. Supporting electrolyte: 0.10 mol L^{-1} PBS + 1.0 mol L^{-1} H_2SO_4. Scan rate: 50 mV s^{-1}.

Parameter Optimization for H_2O_2 Detection

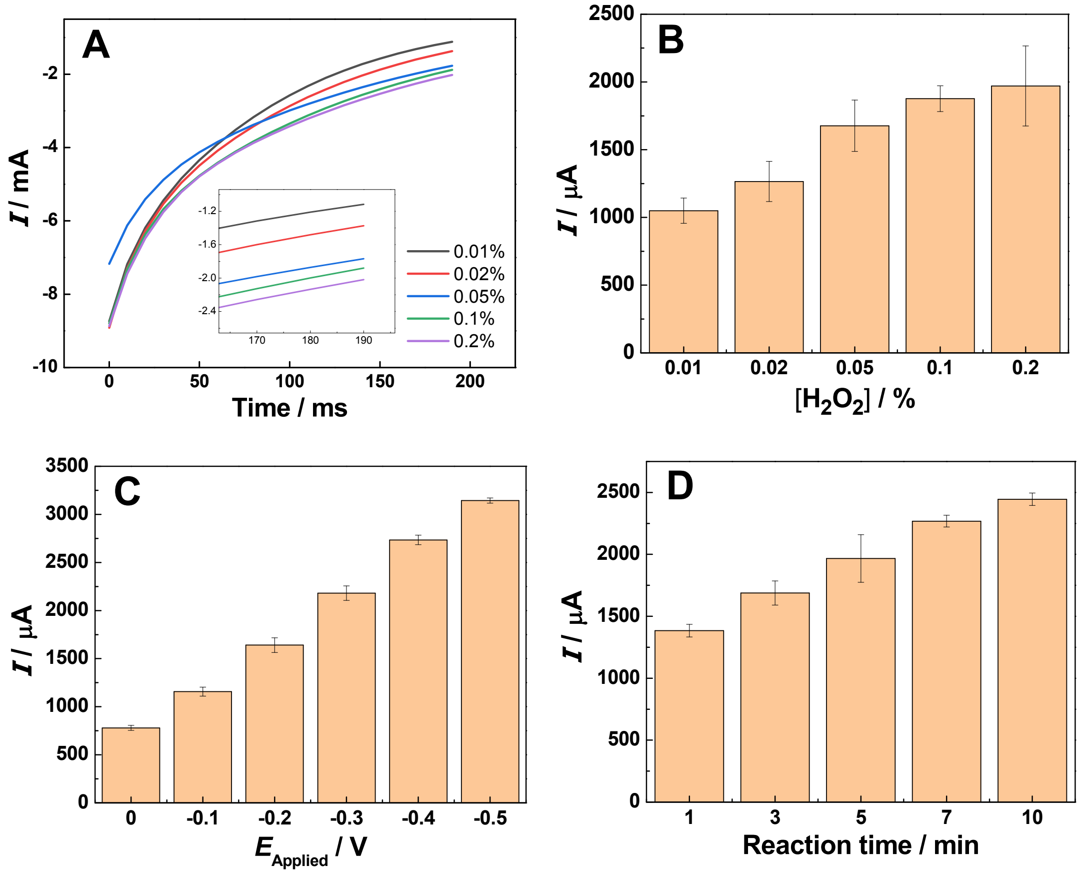

Figure S6. Influence of the (A-B) concentration of H_2O_2, (C) applied potential after 5 min of reaction, and (D) H_2O_2 reaction time. Measurements performed by multiple pulse amperometry (MPA).

Parameter Optimization of the e-PAD immunosensor

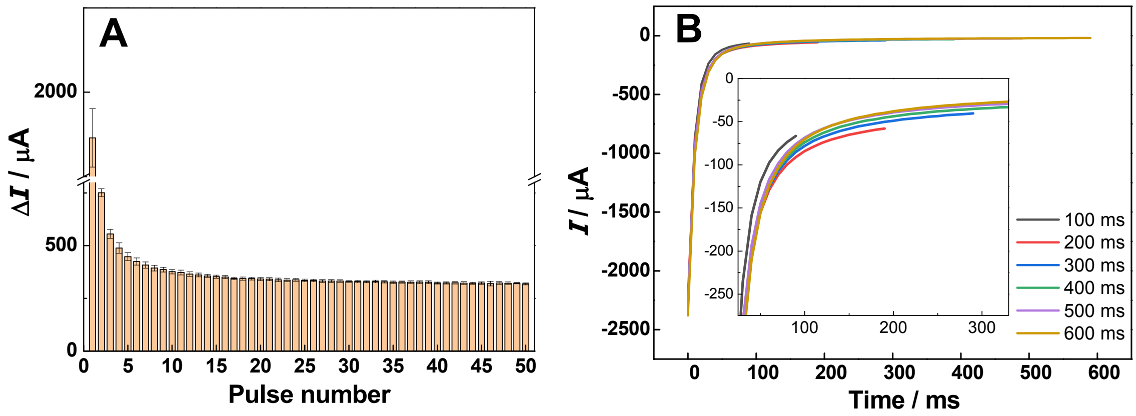

Figure S7. Influence of the (A) pulse number, and (B) pulse time for the measurements performed by multiple pulse amperometry (MPA).

Multivariate Optimization (Factorial Design)

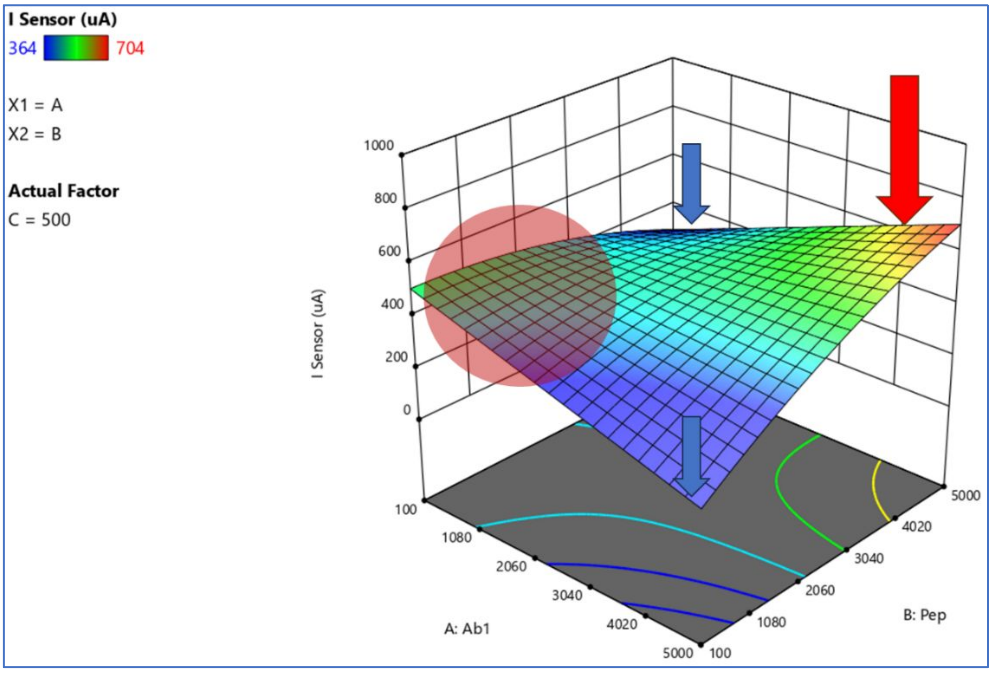

Figure S8. Response surface model obtained for the factorial design, varying the concentrations of capture antibody (Ab1, A axis) and peptide (Pep, B axis) (detection antibody Ab2*, it was fixed in 500 μg mL^{-1}), from 100 to 5000 μg mL^{-1} (100, 500, 1000, 2500, 5000 μg mL^{-1}). Red arrow indicates the best response, and blue arrows indicate the worst response for the combination of Factors 1 and 2. The chosen condition is highlighted by the red circle.

Table S2. ANOVA results obtained using the factorial design.

Source	Sum of Squares	df	Mean Square	F-value	p-value
Block	85094.90	2	42547.45	-	-
**Model**	50987.61	5	10197.52	4.43	0.0141
A-Ab1	6146.92	1	6146.92	2.67	0.1262
B-Pep	378.80	1	378.80	0.1646	0.6916
C-Ab2*	14762.25	1	14762.25	6.41	0.0250
AB	9549.31	1	9549.31	4.15	0.0625
B^2	4144.08	1	4144.08	1.80	0.2026
**Residual**	29918.16	13	2301.40	-	-
Lack of Fit	555.82	1	555.82	0.2272	0.6422
Pure Error	29362.33	12	2446.86	-	-
**Cor Total**	1.660×10^5	20	-	-	-

Table S3. Calibration parameters obtained for the factorial design model.

**Std. Dev.**	47.97	**R^2**	0.6302
**Mean**	551.33	**Adjusted R^2**	0.4880
**C.V. %**	8.70	**Predicted R^2**	0.2948
		**Adeq Precision**	8.4898

Effect of hydrogen peroxide

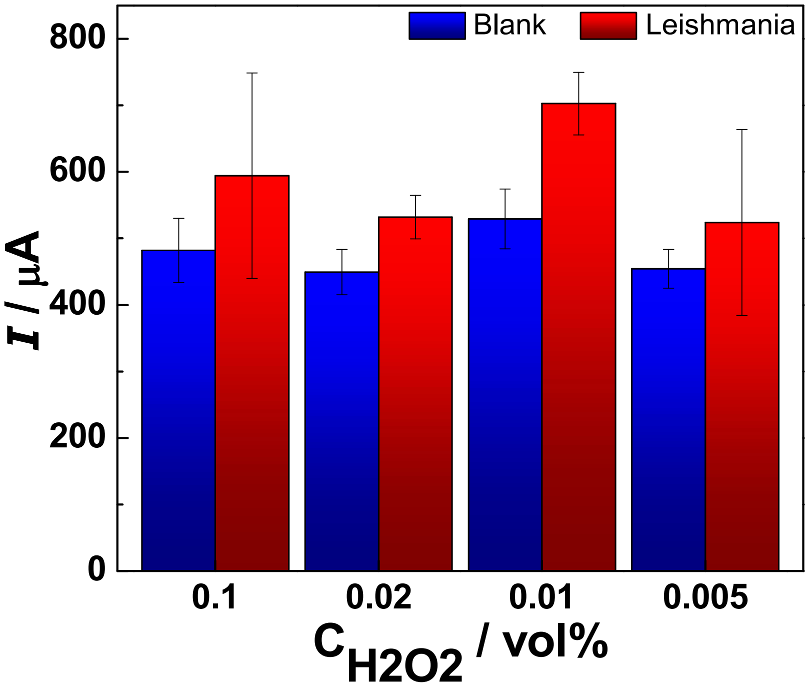

Figure S9. Influence of the H_2O_2 concentration for the detection of *Leishmania amazonensis* peptide (Lap).

Reproducibility of the e-PAD Bioassay

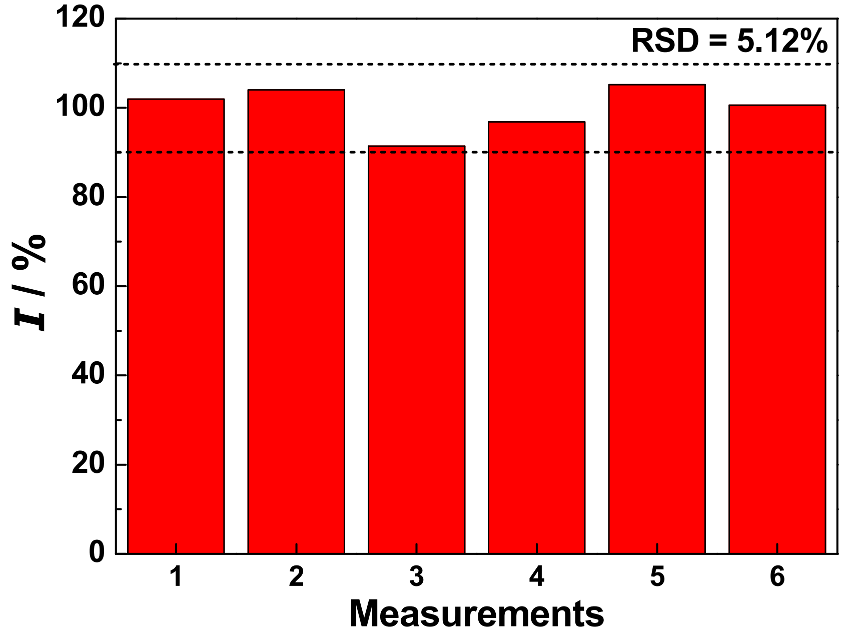

Figure S10. Reproducibility study performed with different e-PADs on different screen-printed electrodes (n = 6).

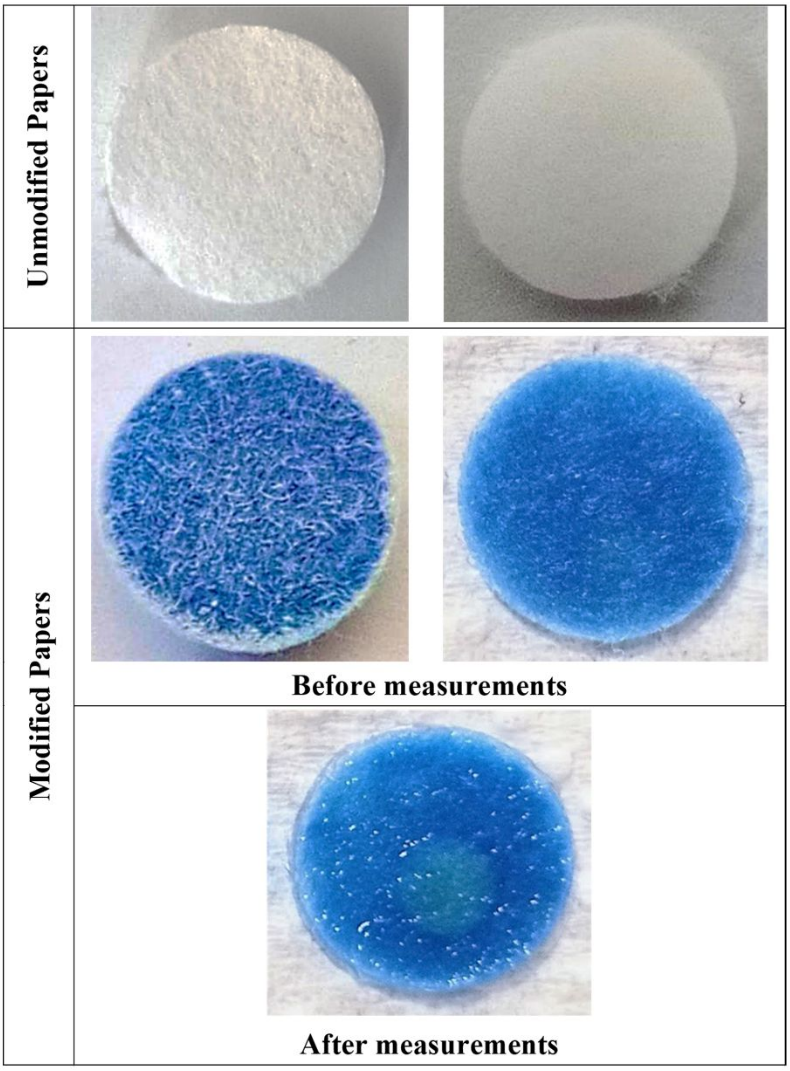

Figure S11. Photographs of the e-PAD. Unmodified paper (white color): dry (left) and wet (right); Modified papers (blue color): Before measurements dry (left, above) and wet (right, above), and after electrochemical measurements (center, below).

Interference evaluation

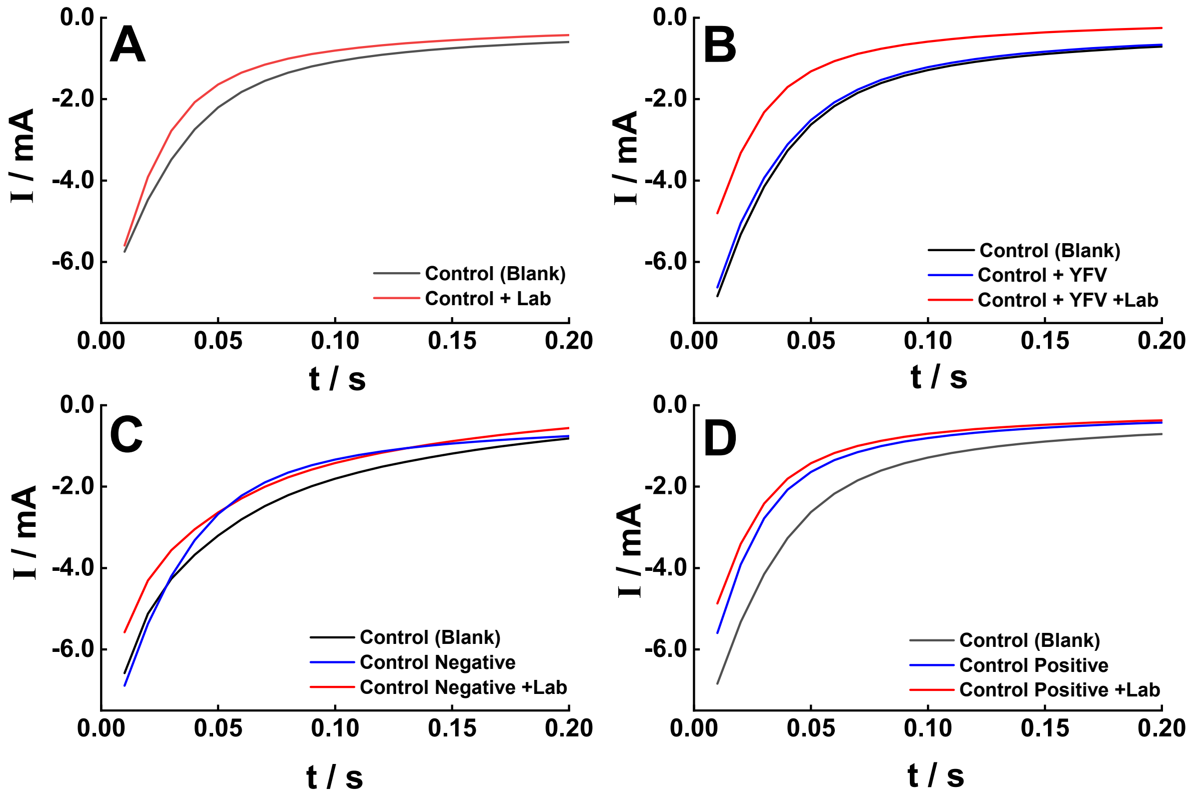

Figure S12. Representative amperometric responses recorded for selectivity analysis in the detection of *L. amazonensis* peptide: (A) target peptide, (B) Yellow Fever Virus (YFV), (C) negative samples, and (D) positive samples as interferents. All measurements were conducted both in the absence (control) and presence (control + Lab) of the target analyte.

Sample Analysis

Table S4. Detection of Leishmania in positive and negative human blood serum samples ($n = 3$, $\alpha = 0.05$).

Sample	t-test value	Student's t table critical value
Positive human blood serum	−3.38<br>−5.06<br>−13.1	2.78 (df = 4)
Negative human blood serum	−1.17<br>−0.704<br>−0.378	
